# Pathogenic Roles of CD14, Galectin-3, and OX40 during Experimental Cerebral Malaria in Mice

**DOI:** 10.1371/journal.pone.0006793

**Published:** 2009-08-27

**Authors:** Miranda S. Oakley, Victoria Majam, Babita Mahajan, Noel Gerald, Vivek Anantharaman, Jerrold M. Ward, Lawrence J. Faucette, Thomas F. McCutchan, Hong Zheng, Masaki Terabe, Jay A. Berzofsky, L. Aravind, Sanjai Kumar

**Affiliations:** 1 Division of Bacterial, Parasitic and Allergenic Products, Center for Biologics and Evaluation Research, Food and Drug Administration, Bethesda, Maryland, United States of America; 2 Division of Emerging and Transfusion Transmitted Diseases, Center for Biologics and Evaluation Research, Food and Drug Administration, Rockville, Maryland, United States of America; 3 National Center for Biotechnology Information, National Library of Medicine, National Institutes of Health, Bethesda, Maryland, United States of America; 4 Infectious Disease Pathogenesis Section, National Institute of Allergy and Infectious Diseases, National Institutes of Health, Rockville, Maryland, United States of America; 5 Laboratory of Malaria and Vector Research, National Institute of Allergy and Infectious Diseases, National Institutes of Health, Rockville, Maryland, United States of America; 6 Vaccine Branch, Center for Cancer Research, National Cancer Institute, National Institutes of Health, Bethesda, Maryland, United States of America; BMSI-A*STAR, Singapore

## Abstract

An in-depth knowledge of the host molecules and biological pathways that contribute towards the pathogenesis of cerebral malaria would help guide the development of novel prognostics and therapeutics. Genome-wide transcriptional profiling of the brain tissue during experimental cerebral malaria (ECM ) caused by *Plasmodium berghei* ANKA parasites in mice, a well established surrogate of human cerebral malaria, has been useful in predicting the functional classes of genes involved and pathways altered during the course of disease. To further understand the contribution of individual genes to the pathogenesis of ECM, we examined the biological relevance of three molecules – CD14, galectin-3, and OX40 that were previously shown to be overexpressed during ECM. We find that CD14 plays a predominant role in the induction of ECM and regulation of parasite density; deletion of the CD14 gene not only prevented the onset of disease in a majority of susceptible mice (only 21% of CD14-deficient compared to 80% of wildtype mice developed ECM, p<0.0004) but also had an ameliorating effect on parasitemia (a 2 fold reduction during the cerebral phase). Furthermore, deletion of the galectin-3 gene in susceptible C57BL/6 mice resulted in partial protection from ECM (47% of galectin-3-deficient versus 93% of wildtype mice developed ECM, p<0.0073). Subsequent adherence assays suggest that galectin-3 induced pathogenesis of ECM is not mediated by the recognition and binding of galectin-3 to *P. berghei* ANKA parasites. A previous study of ECM has demonstrated that brain infiltrating T cells are strongly activated and are CD44^+^CD62L^−^ differentiated memory T cells [1]. We find that OX40, a marker of both T cell activation and memory, is selectively upregulated in the brain during ECM and its distribution among CD4^+^ and CD8^+^ T cells accumulated in the brain vasculature is approximately equal.

## Introduction

Cerebral malaria (CM) is the most severe consequence of a *Plasmodium falciparum* infection and along with severe anemia is a major pathogenic factor behind the approximately 1 million deaths per year, mostly in children aged 2–5 years living in sub-Saharan Africa. In endemic areas, CM has been described as presence of a set of well-defined clinical features with the primary characteristics of unarousable coma, exclusion of encephalopathies, and confirmation of *P. falciparum* infection [2].

A substantial amount of our knowledge regarding the underlying molecular mechanisms that contribute towards the pathogenesis of CM comes from studies using the *P. berghei* ANKA murine model of experimental cerebral malaria (ECM), a well-established surrogate of human CM. In this murine model, depending on the host genetic background, mice can be broadly categorized as susceptible or resistant to ECM. Although infection results in eventual death of all mice, the majority of susceptible mice develop neurological symptoms between days 6–10 post-infection that closely mimic human CM. In contrast, resistant mice (as well as a small portion of susceptible mice) do not exhibit clinical or pathological symptoms of ECM during this 6–10 day window of infection but instead succumb to hyperparasitemia and severe anemia between days 15–21 post-infection.

Several decades of experimental evidence suggest that the clinical manifestations of ECM result from immuno-pathological events that include: 1) the sequestration of mature form parasites in brain capillaries (sequestration hypothesis) [3]; 2) overexpression of inflammatory mediators such as TNF-α (cytokine hypothesis) [4]; and 3) disruption of the blood brain barrier by CD8^+^ T cell mediated apoptosis of endothelial cells (permeability hypothesis) [5], [6]. Recent studies performed in gene deficient mice have confirmed that this complex polygenic trait is influenced by the participation of a growing number of functionally diverse host factors.

T cells play a critical role in the pathogenesis of ECM. This was first recognized when it was reported that athymic nude mice do not develop symptoms of ECM during a *Pb-A* infection [7]. Subsequently, studies based on depletion of T cell subsets in mice and in CD4- and CD8-deficient mice established that both CD4^+^ and CD8^+^ T cells are required for the development of ECM [8], [9]. Importantly, CD4^+^ T cells are critical for the induction of ECM, whereas CD8^+^ T cells are critical during the effector phase of ECM [10]. Accumulating evidence suggests that brain infiltrating T cells during ECM are strongly activated and differentiated memory cells [1].

Previously, we utilized a host genome-wide approach to identify specific alterations in transcription levels by microarray of host genes in mice in the moribund state. After accounting for confounding factors such as mouse genetic background, parasite burden, and disease state (e.g., moribund vs. non-moribund and susceptibility vs. resistance to ECM), we found that more than 200 host genes, based on their transcriptional alterations, were associated with the pathogenic events occurring during ECM [11]. Next we wanted to determine which of the transcriptional altered genes in our dataset were directly involved in the pathogenesis of ECM. The initial criterion for the selection of molecules for further studies was based on functional properties (e.g. cytoadherence, immunological, and inflammatory, etc.) that may suggest a role in the pathogenesis of ECM. These functional properties were ascertained based on earlier published studies and by extensive bioinformatics analyses. Using these criteria, we chose to examine the biological relevance of three molecules - CD14, galectin-3, and OX40 in our dataset that we predicted, based on their function, may play a direct role in the pathogenesis of ECM.

CD14 is a leucine-rich-repeat (LRR) surface protein related to the extracellular LRR-portion of the TLR proteins. It has two well characterized functions that may be pertinent to the pathogenesis of ECM: 1) it is a receptor for lipopolysaccharide of Gram negative bacteria [12] and recognition of microbial ligands by host CD14 often results in the activation of a potent inflammatory cascade, including the release of TNF-α and IL-1β. 2) CD14 is a major receptor involved in the nonphlogistic clearance of cells undergoing apoptosis [13] and its overexpression in the brain may reflect the well-documented increase in endothelial and neuronal apoptotic cells during ECM [6], [14], [15].

Galectin-3 belongs to a large family of animal lectins defined by an evolutionary conserved carbohydrate-recognition-binding domain (CRD), which recognizes β-galactosides. Galectin-3 has a preference for larger oligosaccharides, such as polyNAc-lactosaminoglycan, a polymer of beta (1,3)-linked LacNAc units found on many extracellular matrix and cell surface molecules [16], [17]. Galectin-3 lacks conventional signal peptides and has been localized to the nucleus and cytoplasm [18], [19]. However, it is also secreted by a non-classical pathway and is found on cell surfaces as well as the extracellular matrix. A wide array of functions has previously been assigned to this molecule including the regulation of inflammation [17], [20], apoptosis [21], [22], chemotaxis [23], and cell adhesion [24]. However, given that its preferred target is predominantly extracellular rather than intracellular proteins, it is likely to have a role in interacting with endogenous extracellular glycoproteins or foreign pathogen associated molecular patterns (PAMPs) [25]. Because of these observations and previous reports that galectin-3 can significantly alter the pathogenic course of *Trypanosoma cruzi* [26], *Schistosoma mansoni* [27], *Toxoplasma gondii* [28], and *Leishmania major* [29] protozoan infections, we selected this molecule for further examination of a functional role during ECM.

CD4^+^ and CD8^+^ T cells have been shown to be necessary for the development of ECM [7]–[9]. Therefore, we also investigated the role of the costimulatory molecule OX40 during ECM. OX40, a member of the tumor necrosis factor receptor superfamily [30], is expressed primarily on activated T cells [31] and has been shown to play an important role in the generation of memory [32], [33]. We were particularly interested in measuring the proportion of T cells expressing OX40 because a large portion of brain infiltrating CD4^+^ and CD8^+^ T cells have been shown to be differentiated memory T lymphocytes during ECM [1].

## Results

Infection with *Pb-A* parasites is fatal in 100% of mice. However, the cause of death may vary from ECM to hyperparasitemia and severe anemia depending on the host genetic background. In this study, all *in vivo* murine experiments were performed on the C57BL/6 background, where in our hands, 80–100% of wildtype (WT) mice develop symptoms of ECM.

### CD14-KO mice are protected against ECM and protection is parasitemia dependent

**Figure 1:**
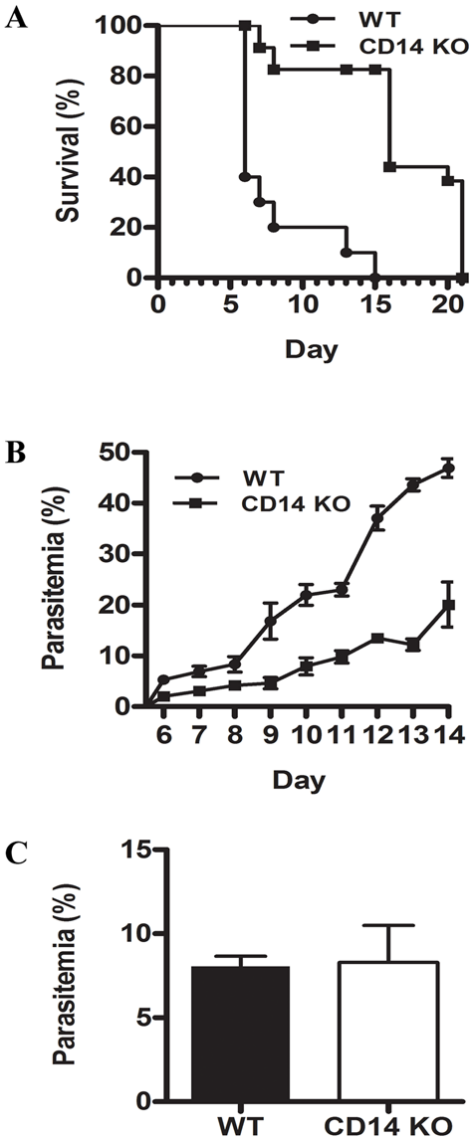
Deletion of CD14 confers protection against ECM and has a negative effect on parasite growth *in vivo*. C57BL/6 WT and CD14-KO mice were challenged with 10^6^ *Pb-A* parasites and susceptibility to ECM was determined based on symptoms exhibited and ability of mice to survive beyond day 10 post-infection. *A:* 16 of 20 (80%) WT mice versus 4 of 19 (21%) CD14-KO mice succumbed to ECM by day 10 post-challenge. *B:* CD14-KO mice that did not develop ECM had approximately 2-fold lower parasite burden between days 6 to 8 and 3.6-fold lower parasite burden between days 9 to 13 post-*Pb-A* infection. *C:* In contrast, WT and CD14-KO mice that developed ECM had no significant differences in parasite burden at onset of ECM.

A correlation between elevated levels of soluble CD14 in serum and complicated *P. falciparum* malaria in humans has previously been reported [34]. In addition, overexpression of CD14 transcription in brain tissue of susceptible mice exhibiting symptoms of ECM has recently been described by our group [11]. To delineate a biological role for CD14 in the pathogenesis of ECM, we measured the susceptibility of CD14-KO mice to ECM. Simultaneously, we also determined if absence of host CD14 had an influence on the *in vivo* growth of intra-erythrocytic parasites which may in turn influence susceptibility to ECM. In cumulative data collected from two independent experiments (n = 10 per group for experiment 1 and 9 CD14-KO and 10 WT for experiment 2), we find that following infection with *Pb-A* parasites, 16 of 20 (80%) WT mice developed ECM (Fig. *1A*) by day 8. In comparison, only 4 of 19 (21%) CD14-KO mice developed symptoms of ECM indicating that loss of CD14 confers a highly significant protection from ECM (p<0.0004, Fisher's exact test). These results strongly indicate that CD14 plays an important role in the expression of ECM. Although protection in CD14-KO mice was highly significant, this protection was incomplete, suggesting that additional host molecules with functionally redundant roles may be able to substitute for CD14 in the pathogenesis of ECM.

Surprisingly, we also find that loss of CD14 had an adverse effect on parasite growth in C57BL/6 mice (p<0.0001, two-way ANOVA). Among mice that did not develop ECM, between days 6 to 8 post-infection, mean parasite burden was approximately 2-fold lower in CD14-KO mice compared to WT mice (4.2% vs. 8.3% on day 6) (Fig. *1B*). The difference in parasitemia was even more drastic (3.6-fold lower) between days 9 (4.6% vs. 16.9%) and 13 (12.2% vs. 43.6%), the post-ECM phase. However, among mice that developed ECM, there was no significant difference in mean parasitemia between WT (8.1±.6% on day 6) and CD14-KO (8.3±2.2% on day 6) mice during the cerebral phase (p = 0.9185, Mann−Whitney test) (Fig. *1C*). Although it is possible that the dual roles of CD14 as a mediator of the pathogenesis of ECM and a positive regulator of parasite density operate independent of each other, our results suggest that CD14-mediated pathogenesis of ECM is directly dependent on the level of parasitemia; CD14-KO mice that were able to overcome resistance to ECM (n = 4) had a parasite burden similar to WT mice.

### Deletion of the *lgals3* gene results in partial protection from ECM

**Figure 2:**
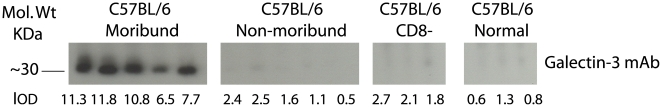
Induction of galectin-3 during experimental cerebral malaria. Expression of galectin-3 was measured in brain tissue samples in individual moribund (n = 5), non-moribund (n = 5), CD8-KO ( = 3), and normal (non-infected, n = 3) C57BL/6 mice by ECL-based western blot analysis. Expression levels were determined based on the intensity of protein bands using Meta1 Morph 6.1 software and are represented as average integrated optical densities (IOD) units. The IOD units shown are values×1000. Details of antibodies and western blot reagents used can be found in the Materials and Methods.

We previously reported that transcription of galectin-3 is induced in brain tissue of susceptible mice that develop ECM and compared to CD8-KO mice which are resistant to ECM, galectin-3 was overexpressed by 6.8 fold in moribund mice [11]. In this study, we quantified the amount of galectin-3 protein in brain tissue of mice during the cerebral phase in order to establish whether galectin-3 expression in the brain is a reliable biomarker of the clinical state of ECM. In an ECL-based, semi-quantitative western blot assay, galectin-3 protein expression (represented in average integrated optical densities [IOD] units) was significantly higher in moribund (9,620±1,058) than in non-moribund (1,620±381), CD8-KO (2,200±265) and normal mice (900±208). Thus, compared to non-moribund and resistant CD8-KO mice, moribund mice exhibited a 5.9 and 4.4 fold increase, respectively in galectin-3 protein expression (Fig. *2*).

**Figure 3:**
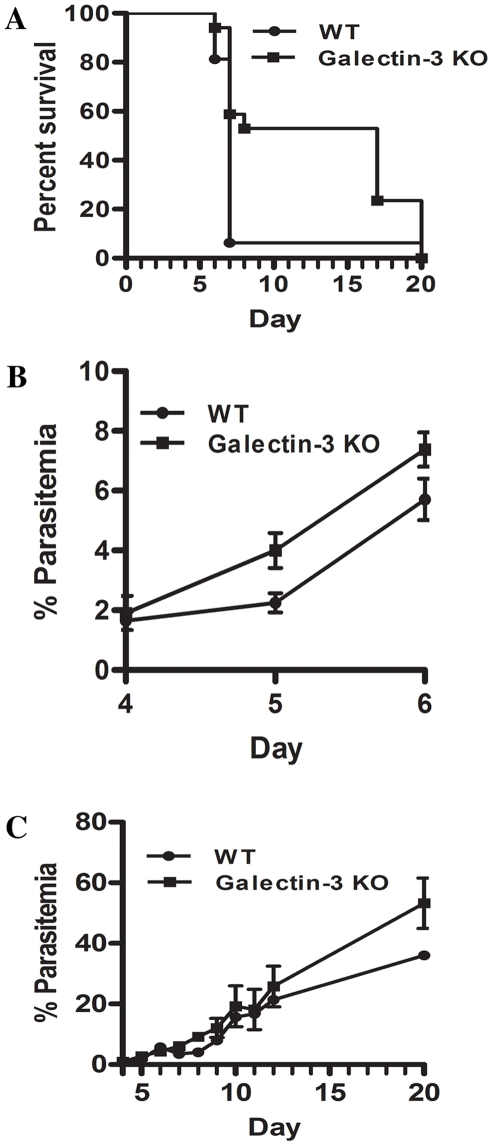
Galectin-3 deficient mice are partially protected against experimental cerebral malaria and developed higher peripheral parasitemia. *A:* Fourteen of 15 (93%) WT mice versus 8 of 17 (47%) galectin-3-KO mice succumbed to ECM by day 8 post-infection. Data shown is cumulated from two independent experiments. *B:* In galectin-3-KO (n = 5) and WT (n = 9) mice that developed ECM, galectin-3-KO mice had moderately higher parasitemia than WT mice (p<0.0137, two-way ANOVA). *C:* Among mice that did not develop ECM, although parasitemia did not differ markedly between days 4 and 12, parasitemia was higher on day 20 in the galectin-3-KO (53.3±8.29%, n = 4) versus WT (36%, n = 1) group.

**Figure 4:**
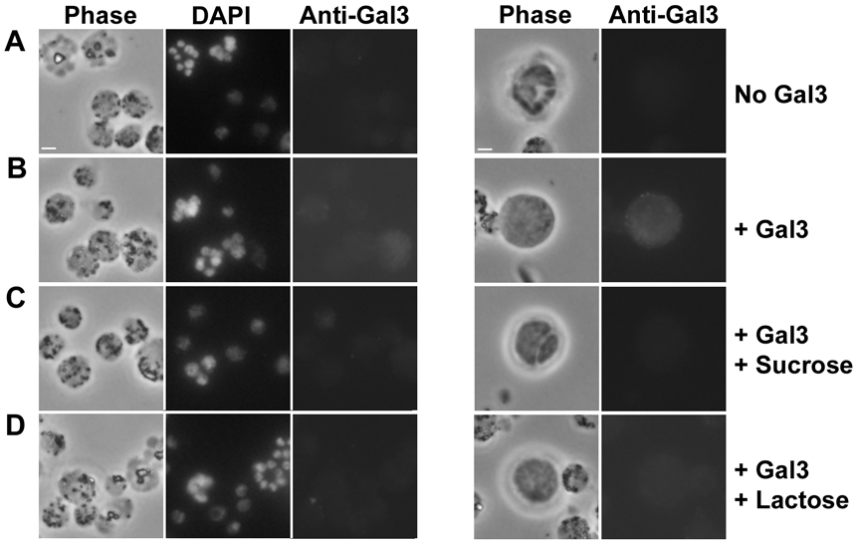
*Pb-A* parasites collected from whole blood at approximately 10% parasitemia were cultured overnight to obtain schizont stage parasites. Parasites were then incubated with *A:* no galectin-3 *B:* galectin-3 *C:* galectin-3 and sucrose and *D:* galectin-3 and lactose and subsequently stained with a goat antibody specific for galectin-3 and a fluorescent donkey antibody specific for goat IgG. Right panels show galectin-3 adherence to mouse lymphocytes. Images were collected on an epifluorescence microscope.

We next measured the susceptibility of galectin-3-KO mice to ECM following infection with *Pb-A* parasites. In a cumulation of two independent experiments, 14 of 15 (93%) WT mice developed ECM (Fig. *3A*) by day 7 post-infection. In comparison, only 8 of 17 galectin-3-KO mice (47%) developed symptoms of ECM by day 8 post-infection, indicating that loss of galectin-3 conferred significant (p<0.0073, Fisher's exact test) protection from ECM. We also determined parasitemia beginning on day 4 post infection. Interestingly, in mice that developed ECM, parasitemia was moderately higher in galectin-3-KO mice compared to WT mice (p<0.0137, two-way ANOVA) (Fig. *3B*). Similarly, among mice that did not develop ECM, although parasitemia did not differ greatly between days 4 and 12, we noted that parasitemia was markedly higher on day 20 in the galectin-3-KO (53.3±8.29%) versus WT (36%) group. Because previous studies have demonstrated an ability of galectin-3 to adhere to *T. cruzi*, *S. mansoni*, and *L. major* protozoan parasites [26], [27], [29], we investigated whether galectin-3 induced pathogenesis of ECM might be mediated by the binding of galectin-3 to *Pb-A* parasites. Therefore, we measured the capacity of galectin-3 to adhere to malaria parasites after incubation of mouse recombinant galectin-3 with *Pb-A* schizonts for 1 h at 37°C. We find that galectin-3 binding to parasites (Fig. *4B*) was not significantly above background levels (Fig. *4A*). We also noticed low levels of galectin-3 binding to mouse lymphocytes. This binding decreased slightly in the presence of sucrose, a control disaccharide that is not recognized by galectin-3 (Fig. *4C*), and lactose, a competitive disaccharide that has a high affinity for galectin-3 (Fig. *4D*), suggesting that the observed adherence of galectin-3 to lymphocytes may be nonspecific.

### Selective expression of OX40 during experimental cerebral malaria

**Figure 5:**
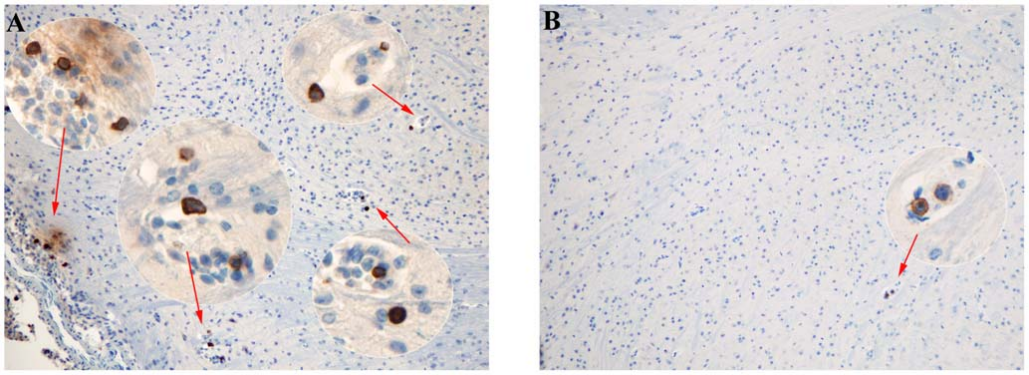
Expression of OX40 in the brain of mice with experimental cerebral malaria. Brain sections from *A:* moribund and *B:* non-moribund *Pb-A* infected mice were stained with a goat antibody specific for mouse OX40 and visualized at 40×magnification. Immunostaining demonstrated strong but highly specific staining of a subset of lymphoid cells within and adjacent to blood vessels in the brains of moribund mice (A) but much fewer positive cells were observed in non-moribund (B) mice.

OX40 is a costimulatory molecule involved in T cell activation and generation of memory. OX40 has previously been implicated in the pathogenesis of several inflammatory diseases such as multiple sclerosis [35], [36], inflammatory bowel disease [37], and rheumatoid arthritis [38], [39]. In our dataset, transcription of OX40 in the brain was increased by approximately two fold in moribund mice compared to non-moribund and resistant mice [11]. We wanted to determine if OX40 protein is preferentially expressed in the brain during ECM. To accomplish this, we performed immunohistological studies in brain sections from C57BL/6 moribund and non-moribund mice. We found that mice displaying symptoms of ECM strongly expressed OX40 on a subset of lymphoid cells in the brain (Fig. *5A*). In comparison, tissue sections from non-moribund mice had much fewer cells expressing OX40 (Fig. *5B*) suggesting that expression of OX40 in the brain during *Pb-A* infection may correlate with disease progression.

**Figure 6:**
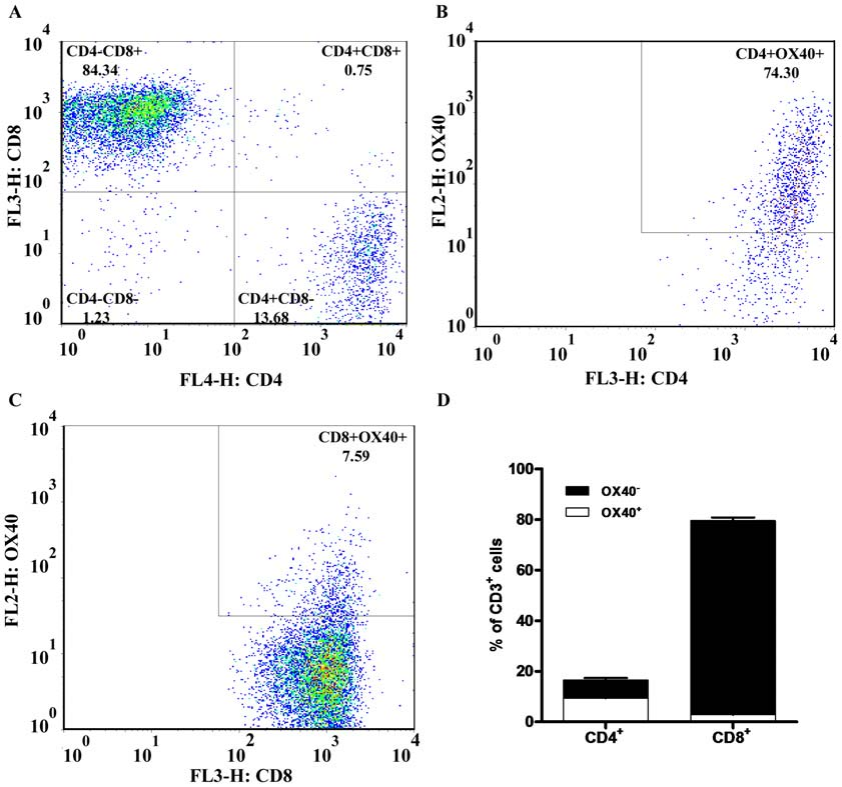
OX40 expression on T cell subsets present in the brain of moribund mice. Total brain leukocytes (sequestered and nonsequestered) were stained with fluorescent-labeled antibodies against TCRβ, CD4, CD8, and OX40. The proportion of *A:* CD4^+^ and CD8^+^ *B:* OX40^+^CD4^+^ and *C:* OX40^+^ CD8^+^ T cells was determined by flow cytometry. *D:* Although OX40 is preferentially expressed on CD4^+^ T cells, due to the comparatively higher number of CD8^+^ T cells in the brain, the absolute number of OX40^+^CD4^+^ and OX40^+^CD8^+^ T cells accumulated in the brain vasculature is approximately the same.

We next performed flow cytometric analysis to determine the percentage of T cells accumulated in the brain vasculature that express OX40 during ECM in mice. In moribund mice, we find that 19.5±2.4% of CD3^+^ brain lymphocytes expressed OX40. Because OX40 is expressed on both CD4^+^ and CD8^+^ T cells and both subsets are known to play an active role in the pathogenesis of ECM, we next wanted to examine the distribution of OX40 among T cell subsets. During the symptomatic effector phase of ECM, we found that 13.68% and 84.34% of CD3^+^ brain lymphocytes were CD4^+^CD8^−^ and CD4^−^CD8^+^ T cells, respectively (Fig. *6A*). However, although there were comparatively fewer CD4^+^ T cells in the brains of mice with ECM, a majority of these CD4^+^ T cells (74.30%) expressed OX40 (Fig. *6B*). Conversely, although 84.34% of CD3^+^ brain lymphocytes were CD8^+^ T cells, only 7.59% of these CD8^+^ T cells expressed OX40 (Fig. *6C*). Consequently, although the proportion of CD4^+^ and CD8^+^ T cell subsets differed greatly in the brain during the effector phase of ECM, the absolute number of CD4^+^OX40^+^ (1184±250) and CD8^+^OX40^+^ (976±388) T cells remained approximately the same (Fig. *6D*). We also measured the expression of OX40 in the spleen of mice with ECM and found that similar to the brain, OX40 was predominantly expressed on CD4^+^ T cells; 42.9% of CD4^+^ versus 3.9% of CD8^+^ splenic T cells expressed OX40 (data not shown).

We also performed flow cytometric analysis of T cell subsets in infected C57BL/6 non-moribund mice. Results showed that non-moribund mice had fewer CD4^+^OX40^+^ (172±138) and CD8^+^OX40^+^ (55±44) T cells in the brain compared to moribund mice. This reduction in OX40^+^ brain lymphocytes is most likely a consequence of the significantly fewer CD3^+^ lymphocytes observed in the brain of non-moribund (1592±800) compared to moribund (13,072±3881) mice.

## Discussion

Based on our microarray dataset, we chose to determine the in-depth role of three molecules in the pathogenesis of ECM in mice. First, our results demonstrate that the CD14 molecule plays a key role as a mediator of the pathogenesis of ECM. In earlier studies, the presence of elevated levels of soluble CD14 in serum from patients with complicated *P. falciparum* malaria has been reported [34]. Consistent with these findings, induction of CD14 transcription in brain tissue of susceptible mice exhibiting symptoms of ECM has recently been described by our group. In this microarray study, transcription of ECM was increased by 2.7±0.1 and 6.2±0.9 fold during ECM compared to BALB/c resistant and C57BL/6 non-moribund mice, respectively [11]. Further investigation in mice deficient for the CD14 gene revealed that loss of CD14 had a dual effect following infection with *Pb-A* parasites. First, CD14-deficient mice were highly resistant to ECM (only 21% of CD14-KO versus 80% of WT mice developed ECM, p<0.0004) (Fig. *1A*) and secondly, loss of ECM associated with a significantly reduced parasite burden throughout the course of *Pb-A* infection in CD14-KO mice (Fig. *1B*). Curiously, our data showing that loss of CD14 molecule associated with protection from ECM is somewhat in conflict with a previous study [40] in which 100% of mice deficient for the CD14 gene developed fatal ECM within 6 to 8 days after infection with 10^6^ *P. berghei* ANKA-parasitized RBCs. The reason behind the inconsistent findings as reported here and in the earlier study is not clear. However, we would like to emphasize that while our results are based on two independent ECM studies that involved a total of 19 CD14-KO mice, the study by Togbe *et al* [40] used only 7 CD14-KO mice in two independent experiments. Although the CD14-KO mice used in our study and the Togbe *et al* study were derived from the same genetic stock [41], it is possible that the CD14-KO strains used in the two studies differ in a subtle way that needs to be further examined. One such subtle difference is that the Togbe *et al* study utilized CD14-KO mice that were backcrossed at least ten times on the C57BL/6 genetic background while the CD14-KO mice used in our study (N_7_F_14_N_1_ mice) were backcrossed eight times. Differences in the *Pb-A* parasite lines could also account for the discrepant results between the two studies. Green fluorescent protein transgenic parasites derived from the cl15cy1 clone of *Pb-A* were used in the Togbe *et al* study while in this study, we used an uncloned line of *Pb-A* parasites that had undergone no genetic manipulation. Additional studies using a large sample size of mice will enhance our knowledge of the role of CD14 in ECM. Nonetheless, we have noted that in agreement with our results, Togbe *et al* also find that compared to WT C57BL/6 mice, CD14-KO mice had a more than two-fold reduction in parasite burden on day 7 post-*Pb-A* infection. In their study, parasite burden was not reported for the entire course of *Pb-A* infection.

CD14 exerts it pro-inflammatory action by acting as an accessory molecule for TLRs [42], [43]. Recent studies have shown that malaria GPI and hemozoin are ligands for TLR2 and TLR9, respectively [44], [45]. However, studies investigating the role of TLR signaling pathways in the pathogenesis of ECM are conflicting. In a study conducted by Coban *et al*, TLR2^−/−^ and TLR9^−/−^ mice, but not TLR4^−/−^, TLR5^−/−^, and TLR7^−/−^ mice, were significantly protected from ECM [46]. Griffith *et al* demonstrated that TLR9^−/−^ mice, but not TLR2^−/−^ and TLR4^−/−^ mice, were partially protected from ECM [47]. In contrast, Togbe *et al*. found that TLR2^−/−^ and TLR9^−/−^ mice, as well as TLR3^−/−^, TLR4^−/−^, TLR6^−/−^, and TLR7^−/−^ mice, were as sensitive to ECM as WT mice [40]. In agreement with Togbe *et al*, a study employing triple knockout mice found that the survival rates of TLR2/4/9^−/−^ mice were comparable to WT mice after *Pb-A* infection [48]. While our study does not differentiate between the TLR-dependent and TLR-independent roles of CD14, on the whole the above considerations are not inconsistent with CD14 acting via the TLR signaling pathways in ECM.

An unexpected result of this study was that loss of CD14 had an adverse effect on parasite growth in C57BL/6 mice. Mean parasite burden was approximately 2-fold lower (days 6 to 8 post-infection) and 3.6-fold lower (days 9 to 13 post-infection) in CD14-KO mice compared to WT mice (Fig. *1B*). Importantly, resistance of CD14-KO mice to ECM was parasitemia dependent; the few CD14-KO mice that developed ECM had a parasite burden comparable to WT mice (Fig. *1C*). Interestingly, this is not the first study to report the ability of CD14-deficient mice to control a pathogen burden more efficiently than WT mice. In a study conducted by Haziot *et al*, CD14-deficient mice were not only resistant to a lethal challenge (5×10^6^ cfu) of *Escherichia coli* 0111:B4, but also had a 27-fold lower level of bacteremia in the blood [49]. This increased clearance of *E. coli* by CD14-deficient mice was attributed to a rapid infiltration of neutrophils (PMNs) in the peritoneal cavity that was significantly delayed in normal mice [50]. It is important to note that neutrophils have been shown to phagocytose and kill malaria parasites *in vitro* [51]–[53]. Nonetheless, further studies are needed to determine the mechanism of CD14 dependent regulation of *Pb-A* parasite density. Regardless of the mechanism, a therapeutic agent that targets CD14 could potentially be useful in preventing CM while simultaneously lowering parasite burden. Importantly, administration of human recombinant soluble CD14 (rsCD14) that might compete with CD14 receptor significantly protected mice from LPS-induced mortality [54].

We have also demonstrated that galectin-3 protein is overexpressed in mice exhibiting symptoms of ECM (Fig. *2*) and deletion of the galectin-3 gene confers partial but significant (p<0.0073) protection from ECM in C57BL/6 mice challenged with *Pb-A* (Fig. *3A*). To our knowledge, this is the first study to address the role of galectin-3 in the pathogenesis of malaria. In previous studies, important and diverse roles have been assigned to galectin-3 during other protozoan parasite infections [26], [27], [28]. Interestingly, in a study evaluating the role of galectin-3 in leishmaniasis, galectin-3 recognized lipophosphoglycan (LPG) of *L. major* but not *L. donovani* and this species-specific recognition of the polygalactose epitope of *L. major* LPG resulted in cleavage of galectin-3 to a truncated form that is incapable of oligomerization, a prerequisite for the immunomodulatory activities of galectin-3 [29]. The authors proposed that truncation of galectin-3 during *L. major* but not *L. donovani* infection may account for differences in pathogenesis between the two species. A similar type of mechanism related to the processing of galectin-3 that is triggered by only a few *Plasmodium* species might be a reason why not all malaria parasites cause the pathogenesis of CM in their respective hosts.

The above mentioned studies and our data provide evidence that galectin-3 can significantly alter the pathogenic course of a parasitic disease. In a majority of these infections, the role of galectin-3 appears to depend on direct interaction with the parasite. However our studies did not find any significant adherence of galectin-3 to schizont stage parasites (Fig. *4B*) above background levels (Fig. *4A*). We speculate that rather than binding parasite moieties, the role of galectin-3 in ECM might result from its binding endogenous oligosaccharides on matrix proteins. Galectin-3 is predominantly expressed in macrophages [23] and might be released upon lysis of brain-infiltrating macrophages. It has been observed that in galectin-3-KO mice, alternative macrophage activation induced by extracellular galectin-3 via IL-4/IL-13 is repressed [55], suggesting that this pathway might be involved in the role of ECM. In light of the results obtained in the leishmaniasis study, we systemically surveyed the galectin-3 gene for SNPs in humans using the HAPMAP data (http://www.hapmap.org). We discovered that this molecule contains one protein-coding SNP (rs4652, A/C) that shows a dramatic difference between sub-Saharan African and non-African populations (96% of chromosomes from the former show the C allele). This particular SNP maps to the N-terminal low-complexity region, which is unique to galectin-3 among members of the galectin family and is required for high avidity binding to multivalent glycoconjugates [56]. This low complexity segment is required for multimerization of galectin-3 [57] and is predicted to adopt a potentially collagen-like structure as a consequence of its repeating pattern of glycines and prolines. Given that the A/C SNP produces a P/T substitution, it could affect the multimerization of galectin-3. Hence, the difference between the sub-Saharan African and non-African populations at this protein position might be a reflection of selection driven by malaria which is prevalent in the former. Thus, despite the only partial level of protection from the *lgals3* gene deletion, it might be useful to further investigate its role as a factor in CM pathogenesis.

We next chose to examine the costimulatory molecule OX40, a marker of both T cell activation and the generation of memory, during ECM. Histological examination of brain sections revealed that mice displaying symptoms of ECM strongly expressed OX40 on a subset of accumulating lymphoid cells in and adjacent to blood vessels in the brain (Fig. *5A*). OX40 was expressed on comparatively fewer cells in brain tissue of non-moribund mice (Fig. *5B*) suggesting that expression of OX40 in the brain may correlate with disease progression. Although OX40 is more commonly expressed on the CD4^+^ subset of T cells, expression has also been observed on CD8^+^ T cells that are strongly activated [58], [59]. We measured expression of OX40 on T cell subsets in the brain by flow cytometry in order to determine whether OX40 is expressed on CD4^+^ or CD8^+^ T lymphocytes. In mice exhibiting symptoms of ECM, 13.68% and 84.34% of CD3^+^ brain lymphocytes were CD4^+^ and CD8^+^ T cells, respectively (Fig. *6A*). However, we found that OX40 was expressed in the majority (74.30%) of CD4^+^ T cells (Fig. *6B*). In contrast, only 7.59% of CD8^+^ T cells co-expressed OX40 (Fig. *6C*), but due to the greater number of CD8^+^ T cells in the brain, the absolute numbers of OX40^+^CD4^+^ and OX40^+^CD8^+^ T cells were similar.

While it is difficult to assess the pathogenic nature of CD4^+^ T cells due to their early role in infection, the symptomatic effector phase of ECM is believed to be precipitated by perforin-mediated apoptosis of brain endothelial cells by pathogenic CD8^+^ T cells [6]. However, it is not known whether these pathogenic CD8^+^ T cells are a heterogeneous population with multiple phenotypes or a small homogeneous population such as the OX40^+^ subset of CD8^+^ T cells accumulated in the brain vasculature characterized in this study. Future studies performed in mice deficient for OX40 will need to be performed to determine whether OX40 is necessary for the pathogenesis of ECM. Furthermore, kinetic analysis by *in vivo* depletion of OX40 during different stages of *Pb-A* infection may be useful in determining which subset of OX40^+^ T cells could potentially be involved in disease.

We believe that by utilizing a combination of approaches including mining the dataset of transcriptionally altered genes that are specifically related to the pathogenesis of ECM and further investigations in gene knockout mice and immunohistology studies, we have identified three novel host molecules – CD14, galectin-3 and OX40 that may play a critical role in the pathogenesis of ECM. Further in depth studies in mice and comparative investigations of young children undergoing clinical symptoms of CM or experiencing asymptomatic malaria during *P. falciparum* infections will be required to firmly establish the role of these molecules in CM.

## Materials and Methods

### Mice and parasite infections

Six to eight week old female wild type (WT), CD14-KO (CD14^−/−^), galectin-3-KO (gal3^−/−^), and CD8-KO (CD8^−/−^) mice on the C57BL/6 background were purchased from The Jackson Laboratory (Bar Harbor, ME). CD14-KO and galectin-3-KO mice were incipient congenic mice backcrossed on the C57BL/6 genetic background eight and six generations, respectively. All mice in these studies were maintained at a National Institute of Allergy and Infectious Diseases or Food and Drug Administration animal care facility and treated in accordance and guidelines of the Animal Care and Use Committee.

*P. berghei* ANKA (*Pb-A*) parasites (an uncloned parasite line) were stored as frozen stabilities in liquid nitrogen at the Laboratory of Malaria and Vector Research, NIAID. Frozen stabilities were injected in donor mice that were compatible with the genetic background of the experimental mice. Subsequently, parasite infection was induced by intraperitoneal injection of 10^6^ *Pb-A* parasites and mice were monitored for clinical symptoms of ECM (hemi or paraplegia, deviation of the head, tendency to roll over upon stimulation, ataxia, and convulsions) beginning on day 3 post-infection. In most instances, parasitemia (parasitized RBCs/total RBCs×100) was enumerated by examining Giemsa-stained thin blood films.

### Statistical analysis

The Fisher's exact test was used to determine differences in survival between WT and CD14-KO and galectin-3-KO mice. Differences in parasitemia were analyzed using two-way ANOVA or the Mann−Whitney test.

### Western blot analysis

To determine whether galectin-3 protein expression is associated with the ECM disease state, expression of galectin-3 protein was measured in whole brain tissue of moribund and non-moribund (infected C57BL/6), resistant (infected C57BL/6 CD8-KO), and normal (uninfected C57BL/6) mice. Brain samples were taken between days 6 and 10 post-infection. Protein samples from brain tissue were prepared as a 10% brain homogenate, and tissue specific galectin-3 protein was detected using antibody specific for human recombinant galectin-3 (Abcam Inc, Cambridge, MA) and a commercially obtained chemiluminescence-linked western blot kit (Western Light Tropix, Bedford, MA). Protein samples were subjected to electrophoresis on a 4-20% Tris-Glycine PAGE gel and then transferred onto a PVDF membrane. Following incubations with the primary and secondary antibodies, protein bands were visualized with ECL detection reagents, and the integrated optical densities (IOD) for each lane were measured using Meta Morph 6.1 Software.

### Immunohistological studies

Tissue sections were prepared from the brains of moribund and non-moribund mice infected with *Pb-A* for immunohistological staining of OX40. Brain samples were isolated from mice on day 6 post-infection. Tissue sections were fixed in formalin and then stained with a goat polyclonal antibody specific for recombinant mouse OX40/*Tnfrsf4* (R&D Systems, Minneapolis, MN). A rabbit anti-goat antibody (Vector Laboratories, Burlingame, CA) was used for the next detection step by following the manufacture's instructions. Hematoxylin was used as the counterstain. Stained sections were examined by light microscopy.

### Binding of *Pb-A* parasites to galectin-3

We investigated whether *Pb-A* infected red cells (IRBC) were able to bind directly to galectin-3. To accomplish this, whole blood was collected from mice infected with *Pb-A* at approximately 10% parasitemia. Parasites were cultured overnight in RPMI with 20% fetal calf serum to obtain schizont stage parasites. IRBC were then fixed in suspension, washed, and resuspended in PBS-0.1% BSA and fixed IRBC were treated with the following combinations: recombinant mouse galectin-3 (8 µg/ml), recombinant mouse galectin-3 (8 µg/ml) and lactose (20 mM), recombinant mouse galectin-3 and sucrose (20 mM), lactose (20 mM) alone, or sucrose (20 mM) alone. Samples were incubated for 1 h at 37°C, washed with PBS-0.1% BSA, fixed, and then resuspended in PBS-3% BSA. To detect the binding of galectin-3 to IRBC, samples were labeled with a goat anti-galectin-3 antibody followed by a fluorescent donkey anti-goat IgG. Images were collected on an epifluorescence microscope.

### Single Nucleotide Polymorphism (SNP) analysis of human galectin-3

To identify potentially relevant SNPs in the genes in this study, we surveyed the NCBI SNP database using the gene ids for the respective genes. All SNPs were collated and checked for significant differences between the 4 principle populations sampled in HAPMAP. The SNPS showing significant population-wise differences were then examined for evidence of changing the coding sequence of the protein or lying next to known transcription regulatory sites. The former set was also assessed for positive selection using data from Wang et al [60].

### Flow cytometry

We performed flow cytometry to determine the percentage of CD3^+^, CD4^+^, CD8^+^ lymphocytes and OX40 expressing lymphocytes in whole brain leukocyte and splenocyte populations taken from mice undergoing clinical symptoms of ECM on day 6 post-infection. Preparation of whole brain leukocytes from unperfused brain tissue was adapted with modifications from a method previously described [61]. Briefly, single cell suspensions of brain tissue were prepared by treatment with DNase-I (28 IU/ml; Sigma- Aldrich) and collagenase (0.5 mg/ml; Sigma- Aldrich) enzymes for 1 hr at 37°C under frequent agitation and trituration. Purification of leukocyte populations was accomplished by centrifugation at 1600×*g* for 20 min on 30% percoll (Sigma-Aldrich) and the gradient layer containing leukocytes was carefully removed. Splenocytes were prepared by using a previously described procedure [62].

Single cell suspensions of brain leukocytes and splenocytes were blocked with anti-CD16-CD32 (BD Biosciences), stained with FITC-anti-TCRβ, APC-anti-CD4, PerCP-anti-CD8, and PE-anti-OX40 (purchased from either BD Biosciences or eBiosciences) in PBS containing 2% fetal calf serum for 30 minutes at 4°C, washed three times, and then analysed on a FACSCalibur flow cytometer using CellQuest (BD Biosciences) and Flowjo (Treestar) software.
